# Differential modulation of tomato root exudates by *Streptomyces* strains underlies contrasting control of *Fusarium oxysporum* f. sp. *lycopersici*

**DOI:** 10.3389/fpls.2026.1759226

**Published:** 2026-03-04

**Authors:** Valerio Mattei, Kjell Sergeant, Marco Saracchi, Daniela Bulgari, Andrea Kunova, Cristina Pizzatti, Paolo Cortesi, Jenny Renaut, Matias Pasquali

**Affiliations:** 1 Department of Food, Environmental and Nutritional Sciences (DeFENS), University of Milan, Milan, Italy; 2 Luxembourg Institute of Science and Technology (LIST), Belvaux, Luxembourg

**Keywords:** bacteria plant interactions, biological control, root exudate, chemotropism, *Streptomyces* - tomato - fungi, untarget metabolomics, LC-MS/MS

## Abstract

**Introduction:**

Rhizosphere microbiome is affected and modulated by the complex mixtures of bioactive molecules that are released by plant roots. In this work, two promising plant growth-promoting strains of *Streptomyces* spp. (DEF17 and DEF19) were evaluated for their capacity to modulate tomato roots and exudates metabolic profile and influence *Fusarium oxysporum* f. sp. *lycopersici* (Fol).

**Methods:**

Dual culture assays, chemotropism assays, and *in planta* pathogenesis assays were performed to evaluate the capability of the strains to inhibit Fol growth, repel Fol conidia, and induce plant defense mechanisms both *in vitro* and *in vivo*. Finally, untargeted LC-MS/MS analysis was performed to understand which metabolites are produced and released by tomato roots after plant-bacteria interaction occurs.

**Results:**

In dual culture assays, DEF19 inhibited Fol growth, whereas DEF17 did not. On the other hand, exudates derived from DEF17-seed treated plant showed to be more acidic, thus inducing Fol phobotaxis compared to both DEF19 and control plants. Also, DEF17-seed treated plants significantly reduced disease severity in planta. Metabolomics showed strain- and compartment- specific remodeling with DEF19 affecting it mostly. On the other hand, DEF17 showed a distinct exudate fingerprint enriched in g-glutamyl dipeptides and phenylacetic-acid conjugates, with an interaction-induced differential glycosylation of 2,4-di-tert-butylphenol.

**Discussion:**

Together, these results indicated that tomato plant protection against Fol is consistent with DEF17 through exudate-mediated modulation, highlighting a gap between *in vitro* antagonism and *in planta* efficacy.

## 1 Introduction

Soilborne infections, such as *Fusarium oxysporum* f. sp. *lycopersici* (Fol), pose an important threat to tomato (*Solanum lycopersicum*) cultivation worldwide. Fol is a vascular wilt pathogen that may persist in the soil for years and infect tomato plants through their roots, resulting in significant crop losses (Dean et al., 2012; Michielse and Rep, 2009). Fol infections are especially difficult to treat because the pathogen can persist without the host, and chemical fungicides are not very effective in the field (Corkley et al., 2022; Panth et al., 2020). Thus, there is increasing interest in investigating biological alternatives, like using beneficial microorganisms to improve plant resilience and reduce disease severity in a sustainable manner (Liu et al., 2017).

Among the microbial taxa with potential biocontrol activity, *Streptomyces* species have been widely investigated for their ability to modulate plant physiology, thereby reducing the prevalence and severity of plant diseases (Viaene et al., 2016). These actinomycetes are well known for their complex secondary metabolism, which includes the synthesis of siderophores, antibiotics, volatile organic compounds (VOCs), and enzymes capable of breaking down fungal cell walls (Barka et al., 2016). Moreover, *Streptomyces* strains have also been demonstrated to colonize root surfaces, increase nutrient uptake, and alter the plant hormonal balance in addition to direct antifungal activity (Vurukonda et al., 2018; Colombo et al., 2019; Mattei et al., 2022).

Root exudates play a critical role in the interaction between plants and soil microorganisms. Many primary and specialized metabolites, including sugars, amino acids, organic acids, phenolics, flavonoids, and terpenoids, are present in these exudates and function as signaling molecules as well as sources of nutrients (Lombardi et al., 2018; Canarini et al., 2019; Vives-Peris et al., 2020; Upadhyay et al., 2022). Furthermore, the chemical diversity of root exudates is complex and dynamic, affected by the plant’s developmental stage, nutritional status, and environmental cues, including the presence of bacteria (Korenblum et al., 2020; Mahmud et al., 2021). Plant growth-promoting rhizobacteria (PGPR), including *Streptomyces*, can alter the root exudate profile, which in turn may shape the surrounding microbiota or influence the behavior of pathogens (Wankhade et al., 2025). In the case of Fol, previous studies have demonstrated that its conidia can respond chemotropically to chemical signals released by roots (Turrà et al., 2015), indicating that exudate composition influences the early stages of pathogen recognition and infection.

In this study, we investigated the effects of two different *Streptomyces* strains., DEF17 and DEF19, on the metabolic composition of tomato roots and exudates. DEF17 was taxonomically assigned to *Streptomyces hydrogenans*, whereas DEF19 is referred to as *Streptomyces* sp. Based on previous experiments carried out in our laboratory, *Streptomyces hydrogenans* DEF17 was selected for its ability to produce siderophores (Colombo et al., 2019), while *Streptomyces* sp. DEF19 was selected for its ability to produce chitinases in *in vitro* assays (Colombo et al., 2019). In this regard, using a combination of *in vitro* dual culture assays, fungal chemotropism tests, and untargeted LC-MS/MS metabolomics, we aimed to assess whether microbial seed treatments induce changes in root-associated metabolite profiles and whether these changes correlate with altered behavior of Fol conidia. This integrative strategy allowed us to explore how *Streptomyces* spp. may shape the chemical environment of tomato roots, providing new insights into the functional specificity of different *Streptomyces* strains.

## 2 Materials and methods

### 2.1 Bacterial strains preparation and seed treatment

Using a sterile plastic spatula and 10 mL of sterile distilled water, both *Streptomyces* spp. DEF17 and DEF19 spores were collected from the surface of three-week-old cultures cultured on Czapek’s yeast extract agar (CZY) plates at 25 °C (Mattei et al., 2022; Colombo et al., 2019). After soaking tomato seeds (*Solanum lycopersicum* “Moneymaker”) in 1% sodium hypochlorite for three minutes, the seeds were rinsed with sterile distilled water until disinfectant elimination. Following surface sterilization, the seeds were separated into three groups: a control group that was prepared by soaking seeds in sterile deionized water, and two treatment groups, which were prepared by soaking the seeds in either DEF17 or DEF19 spore solutions (1 × 10^7^ CFU/mL). After treatment, seeds were left under laminar hood flow until completely dried. A total of 20 seeds per treatment were prepared.

### 2.2 Roots and root exudates collection

To avoid cross-contamination, each seed was seeded separately in 50 mL Falcon tubes filled with 5 mL of ½ Murashige and Skoog (MS) medium containing 0.2% agar and grown in growth chamber under controlled environment with a photoperiod of 16 h light/8 h dark, with a photosynthetically active radiation (PAR) of approximately 130 μmol m^-2^ s^-1^ and temperatures of 24 °C/18 °C. Seedlings were harvested, and the roots were cut after 21 days of sterile growth. The residual medium was centrifuged for 10 minutes at 4 °C at 10,000 g. The supernatant, containing root exudates, was then recovered and used for chemotropism assays and LC-MS/MS analysis.

### 2.3 Root and roots exudates metabolites extraction

The root exudates were analyzed without further sample preparation. The roots were dried under vacuum and weighed, and 3 inox marbles were added for grinding the roots in a bead grinder for 2 minutes. To the ground samples extraction solvent (70% ethanol/MQ) was added in a ratio of 1/100 w/v, and the samples were sonicated for 5 minutes at room temperature and subsequently incubated for 1 hour at 25 °C in a thermo block with 1400 g agitation. After centrifugation (4 °C, 20000*g* for 20 minutes) 100 µL was recovered and dried under vacuum. The thus dried extract was resolubilized in 100 µL 20% MeOH/MQ.

### 2.4 LC-MS/MS analysis

All samples were filtered through a PTFE syringe filter (0.22 µm, Millex-LG, Merck KGaA, Germany) and analyzed with LC-MS/MS, as previously described (Halime et al., 2025). Ten microliters of the sample were injected and separated with an Acquity UPLC I-Class system equipped with a diode array detector using a reversed-phase Acquity UPLC BEH C18 column (2.1 × 100 mm, 1.7 μm; Waters, USA). The column was maintained at 50 °C, and a flow rate of 0.5 mL/min was used. The mobile phase consisted of 0.1% (v/v) formic acid in water (A) and 0.1% (v/v) formic acid in acetonitrile (B), with the following gradient: 0 min, 1% B; 4 min, 1% B; 16 min, 5% B; 35 min, 40% B; 45 min, 100% B; 50 min, 100% B; 54 min, 1% B; and 60 min, 1% B.

The UPLC was coupled to a TripleTOF 6600+ mass spectrometer (SCIEX, USA) in positive and negative ionization modes. Electrospray ionization was performed using the following parameters: source temperature 650 °C; ion spray voltage of 4.5 and -4.5 kV for positive and negative mode. The curtain gas (nitrogen) was at 30 psi, and nebulizer and turbine gas (air) at 55 and 50 psi. The declustering potential was set at 60 V in positive and -60 V in negative mode. Survey scans of 175 ms were acquired for information-dependent acquisition. The ten highest single-charged MS ions with an intensity higher than 100 counts/sec were selected for fragmentation. MS/MS spectra were collected in high sensitivity mode with an accumulation time of 200 ms. A sweeping collision energy of 15 eV was applied to all precursor ions for collision-induced dissociation. The dynamic exclusion was set for 2 s after three occurrences before the precursor could be fragmented again.

Progenesis QI (v2.3, Waters) was used for identification and relative quantification; raw data files were aligned, normalized, and relative quantitative analysis was performed based on treatments. The two sample types were analyzed in separate experiments. Features without MS/MS data or known contaminants were omitted for the identification stage. Initial identification relied on the use of an in-house database containing MS, MS/MS and metadata on all identifications obtained in the group. For compounds not present in this in-house database, identification was achieved through MS/MS matching of experimental spectra with spectra found in databases such as GNPS (https://gnps.ucsd.edu/ProteoSAFe/libraries.jsp), MZCloud™ (https://beta.mzcloud.org/), LipidMaps (https://www.lipidmaps.org/), and PubChem (https://pubchem.ncbi.nlm.nih.gov) or available in literature. All accepted identifications were manually validated, and accepted identifications were incorporated into the in-house database for future dereplication. The reported identifications are level 2 identifications as defined by the Metabolomics Standards Initiative.

### 2.5 Fungal culture and conidia preparation

*Fusarium oxysporum* f. sp. *lycopersici*, sequenced strain FOL 4287 (NRRL 34936/CBS 123668/FGSC 9935) was used in all experiments. Fol was cultured on potato dextrose agar (PDA; Difco, USA) and incubated at 24 °C for 7 days. For conidia production, a carboxymethyl cellulose (CMC) medium was prepared based on a protocol adapted from Colombo et al. (2019). The CMC medium consisted of 15 g/L carboxymethyl-cellulose (Sigma-Aldrich, USA), 1 g/L NH_4_NO_3_, 1 g/L KH_2_PO_4_, 0.5 g/L MgSO_4_·7H_2_O (Carlo Erba Reagents, Italy), and 1 g/L yeast extract (Difco Laboratories, USA), adjusted to pH 6.5. After autoclaving, 100 mL of sterile CMC medium was poured into sterilized 250 mL Erlenmeyer flasks and inoculated with six 0.6 cm-diameter plugs of Fol mycelium previously grown on PDA. Cultures were incubated at 24 °C on a rotary shaker for 5 days. After incubation, cultures were filtered through a single layer of Miracloth (Calbiochem, USA) into sterile 50 mL Falcon tubes. Conidia were harvested by centrifugation at 9,500 g for 10 min at 4 °C. The pellet was washed three times with sterile MQ and finally resuspended in 0.01% (v/v) Tween 20 solution. Conidia were counted using a Bürker chamber and adjusted to a final concentration of 5 × 10^6^ conidia/mL for further experiments.

### 2.6 Dual cultures and chemotropism assays

A dual-culture method was used to test DEF17 and DEF19 antifungal activity against *Fol* growth following the protocol from Kunova et al. (2016). Briefly, strain DEF17 and DEF19 were symmetrically streaked on PDA plates, 2.5 cm from the center of the *Fol* mycelial plugs (d = 5 mm). As controls, PDA plates that had only been inoculated with fungal plugs were employed. Three replications of each treatment were carried out. For five days, all plates were incubated at 26 °C. Each *Fol* colony’s diameter was measured, and the inhibition rate (IR) was computed using the formula below reported in Zhao et al., 2022:


IR=[(R1−R2)÷R1] × 100

where R1 = Diameter of pathogenic fungus in the control plate. R2 = Diameter of the pathogenic fungus interacting with the antagonist. Dual culture experiments were repeated in triplicate.

The same exudates used to analyze the tomato metabolomic responses were also used to assess the chemotropic response of Fol conidia to root exudates following the method described by Turrà et al. (2015) with modifications. A 0.5% water agar (WA0.5%) solution was prepared and maintained at 40 °C. Conidial suspensions (5 × 10^6^ conidia/mL) were embedded in 5 mL of WA0.5% to a final concentration of 5 × 10^4^ conidia/mL and poured into standard Petri dishes. After solidification, two wells spaced 1 cm apart were created in the agar using a sterile pipette tip. One well was filled with 50 µL of root exudates from *Streptomyces*-treated plants (DEF17 or DEF19), and the opposing well with 50 µL of control exudates from untreated plants. Plates were incubated at 24°C for 16 hours in the dark. Microscopic evaluation was performed using an optical microscope (200× magnification) to assess the germ tube emergence direction. For each condition, at least 100 hyphal tips were scored. Chemotropic responses were expressed as the proportion of hyphae orienting toward the treatment well (% toward treatment well) versus the control well. Values >50% indicate chemoattraction, while<50% indicate repulsion. Seven independent batches of cells (n = 100 hyphal tips per batch) were scored for each sample. Finally, the pH of the exudates was measured with a pH meter (XS instruments, Italy).

### 2.7 *In planta* pathogenesis assay

To assess the biocontrol activity of both DEF17 and DEF19 *in planta*, a Fol infection assay was done by sowing a total of 60 tomato seeds. Twenty plants per treatment were prepared (DEF17, DEF19 and Control). Seed treatment was done by soaking the tomato seeds in DEF17 or DEF19 spore solution (1x10^7^ spores/ml) or deionized water (control). Tomato plants were grown in a growth chamber, with a 16-h light (∼PPFD of 600 μmol of photons/(m_2_ s_−1_)) and an 8-h dark photoperiod. During the experiment, the recorded average temperatures during the light and dark periods were 28 °C and 22 °C, respectively. The relative humidity (HR) percentage averaged between 74.3% and 51.7% during the dark and light periods, respectively. *Solanum lycopersicum* ‘Moneymaker’, which is susceptible to FOL, was grown in pots of 7 cm × 7 cm x 10 cm, sowing one seed per pot. Plants were grown in a blend (1:1 ratio) of Irish and enriched-black peat-based growth substrate (SER CA-V7 and SER V10-14P, Vigorplant, Italy), previously sterilized for 3 weeks at 55 °C. Pots were randomly distributed and watered every two days with tap water. After 10 days, half of the pots (n=30) were soil drenched with 8 ml of Fol conidia (10^6^ conidia/mL). Disease severity was visually assessed every week for 30 days, starting two weeks after inoculation and following Marlatt (1996) protocol. Briefly, each plant was given a final disease rating on a 1–5 scale: 1 = no visible symptoms; 2 = mild chlorosis with slight wilting or stunting; 3 = moderate chlorosis with noticeable wilting or stunting; 4 = severe chlorosis with strong wilting or stunting; and 5 = plant death. Final assessment was carried out 30 days after inoculation.

### 2.8 Statistical analysis

One-way ANOVA was used on the data, and the Tukey *post-hoc* test (P<0.05) was employed to evaluate the mean differences. GraphPad Prism version 10 for macOS (GraphPad Software, La Jolla, California, USA, www.graphpad.com) was used to conduct the analyses. The captions of each figure and table provide more details.

Moreover, Metaboanalyst v.6.0 (https://www.metaboanalyst.ca) was used to perform multivariate analysis of metabolomics data. Before the analysis, the data underwent sum-normalization, square root transformation, and Pareto scaling. The hierarchical clustering was obtained with the following options: Euclidean distance measure and Ward clustering algorithm. Using a variable importance in projection (VIP) plot, which was produced by a partial least squares discriminant analysis (PLS-DA), the most significant variables (metabolites) linked to the variations between clusters were chosen. VIP > 2 variables are indicated, and a one-way ANOVA was used to determine their significance (p< 0.05).

All experiments were performed using at least three biological replicates unless otherwise stated.

## 3 Results

### 3.1 Dual culture and chemotropism assays

**Figure 1:**
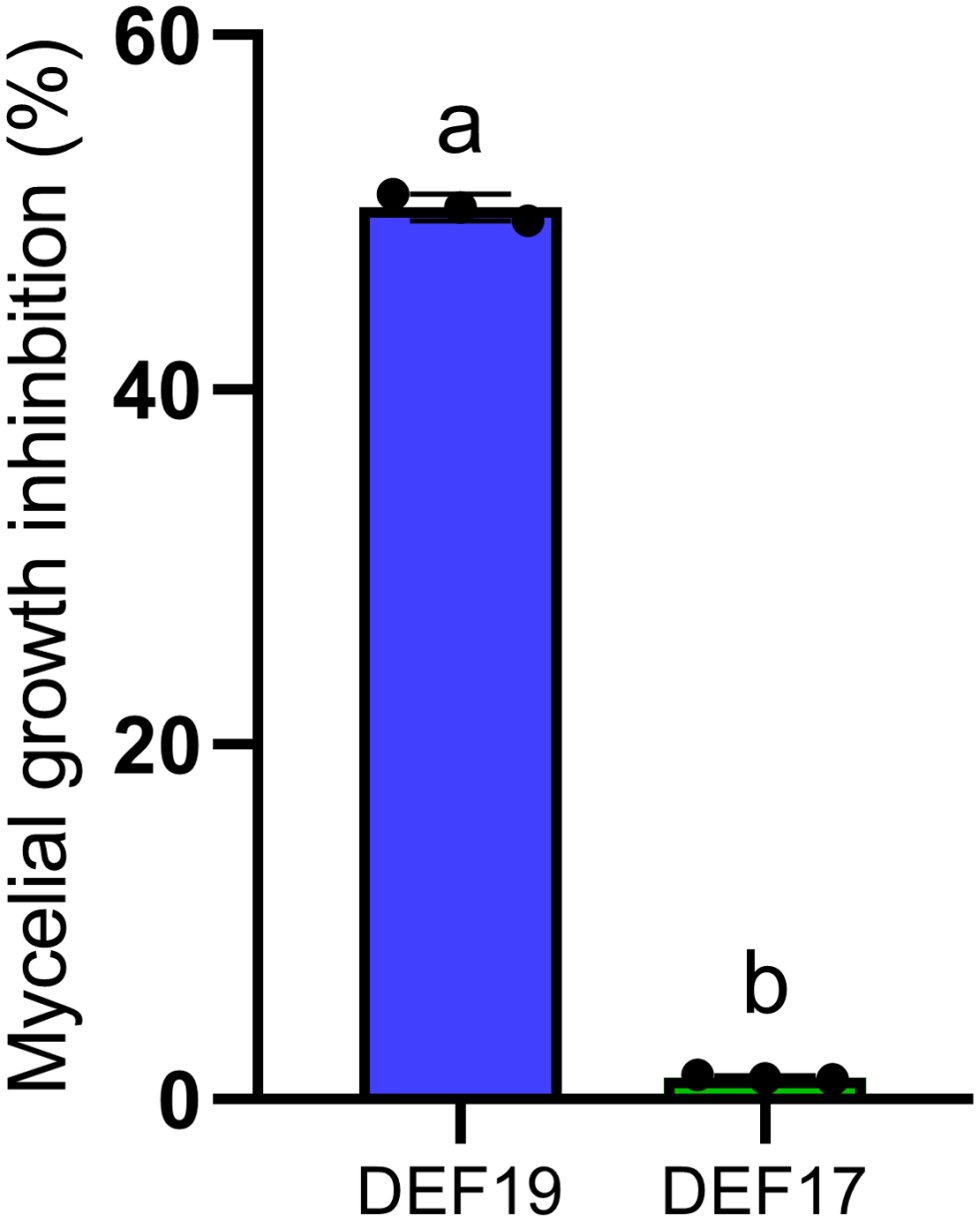
Fungal growth inhibition (%) derived from the application of. DEF17 and DEF19 against Fol *in vitro*. Bars indicate standard error. The letters indicate statistically significant differences (Tukey’s HSD, P< 0.05).

In dual culture assays, DEF19 limited the fungal growth by 50% compared to the control, whereas DEF17 did not show any inhibition (**Figure 1**; **Supplementary Figure S1**).

**Figure 2:**
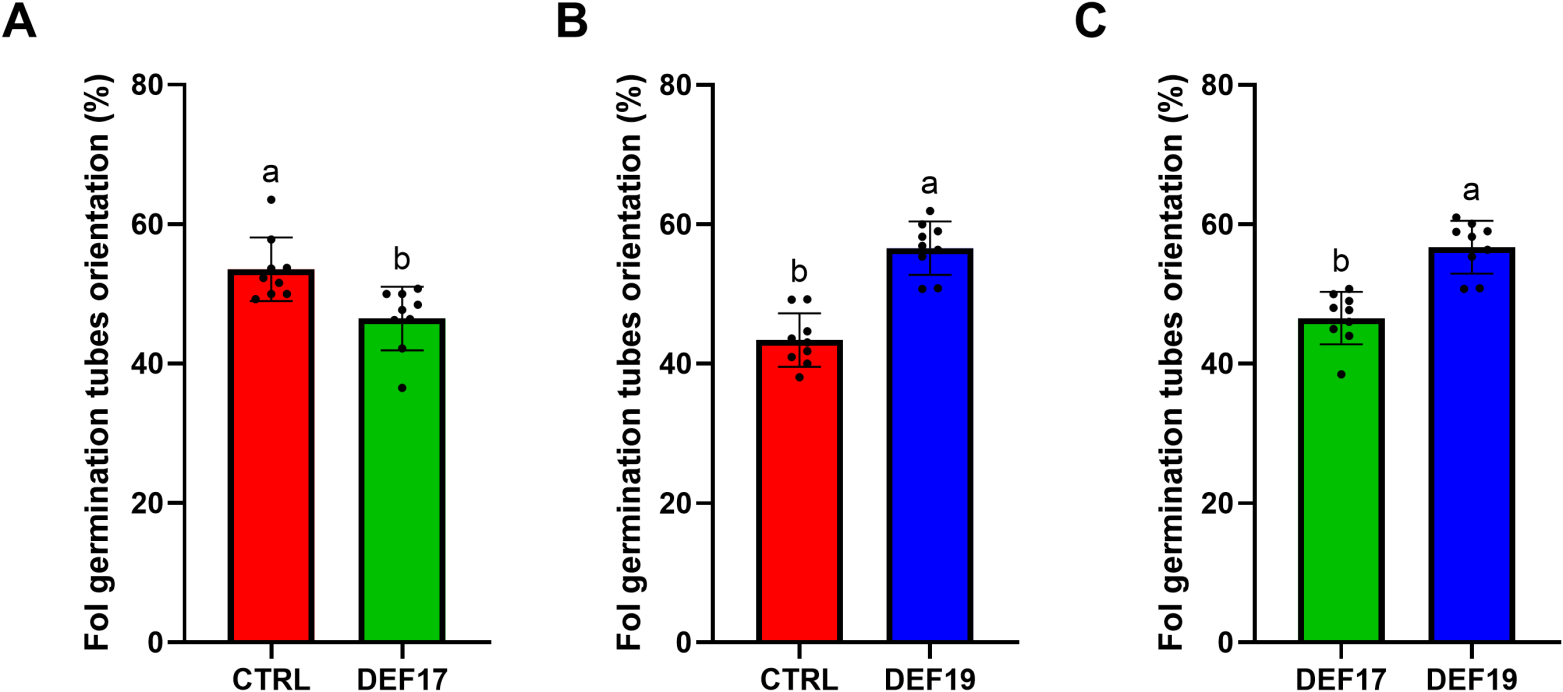
Chemotropism assays indicating the percentage of conidia germination tubes growing towards test sample exudates (DEF17 and DEF19) and control exudates (CTRL). **(A)** Germination tubes preferred direction between CTRL and DEF17; **(B)** Germination tubes preferred direction between CTRL and DEF19; **(C)** Germination tubes preferred direction between DEF17 and DEF19. Letters indicate statistically significant differences (Tukey’s HSD, P< 0.05) Metric shown is ‘% toward treatment well’; >50% = attraction,<50% = phobotaxis.

However, Fol conidia showed different chemotaxis behaviors when exposed to the root exudates obtained from different treatments (**Figure 2**). Exposure of Fol to root exudates of tomato plants grown after inoculation with DEF17 resulted in a significant change in the orientation of Fol conodia hyphal tips. In this regard, 46% of Fol germination tubes grew oriented towards exudates of tomato incubated with DEF17 on average across different replicas compared to both control (**Figure 2a**) and DEF19 (**Figure 2c**). In addition, a shift in the orientation of the conidia grew germination tube was also observed when Fol conidia were exposed to control and DEF19 plant exudates. In fact, 43% and 57% were oriented towards control and DEF19-treated plant exudates, respectively (**Figure 2b**). Furthermore, DEF17 seed-treated plant exudates showed the lowest pH (pH 4.2) compared to both control (pH 4.5) and DEF19-treated plant exudates (pH 4.6), respectively. In this regard, these results highlight the complexity of screening biocontrol agents (BCAs) efficacy using *in vitro* assays.

### 3.2 Untargeted metabolomics analysis

Untargeted LC-MS/MS analysis was performed on root tissues and root exudates of tomato seedlings treated with DEF17, DEF19, and an untreated control. A total of 4272 LC-MS features were detected in root tissues, of which 172 were annotated. Similarly, 4296 LC-MS features were detected in root exudates, of which 104 were annotated. However, after polishing datasets from repeated metabolite hits and selecting only significant metabolites (p value > 0.05), a total of 123 and 81 metabolites were identified for root tissues and exudates, respectively (**Supplementary Table S1**). Representative base peak chromatograms of control, DEF17- and DEF19-treated tomato exudates are provided in **Supplementary Figure S3**.

**Figure 3:**
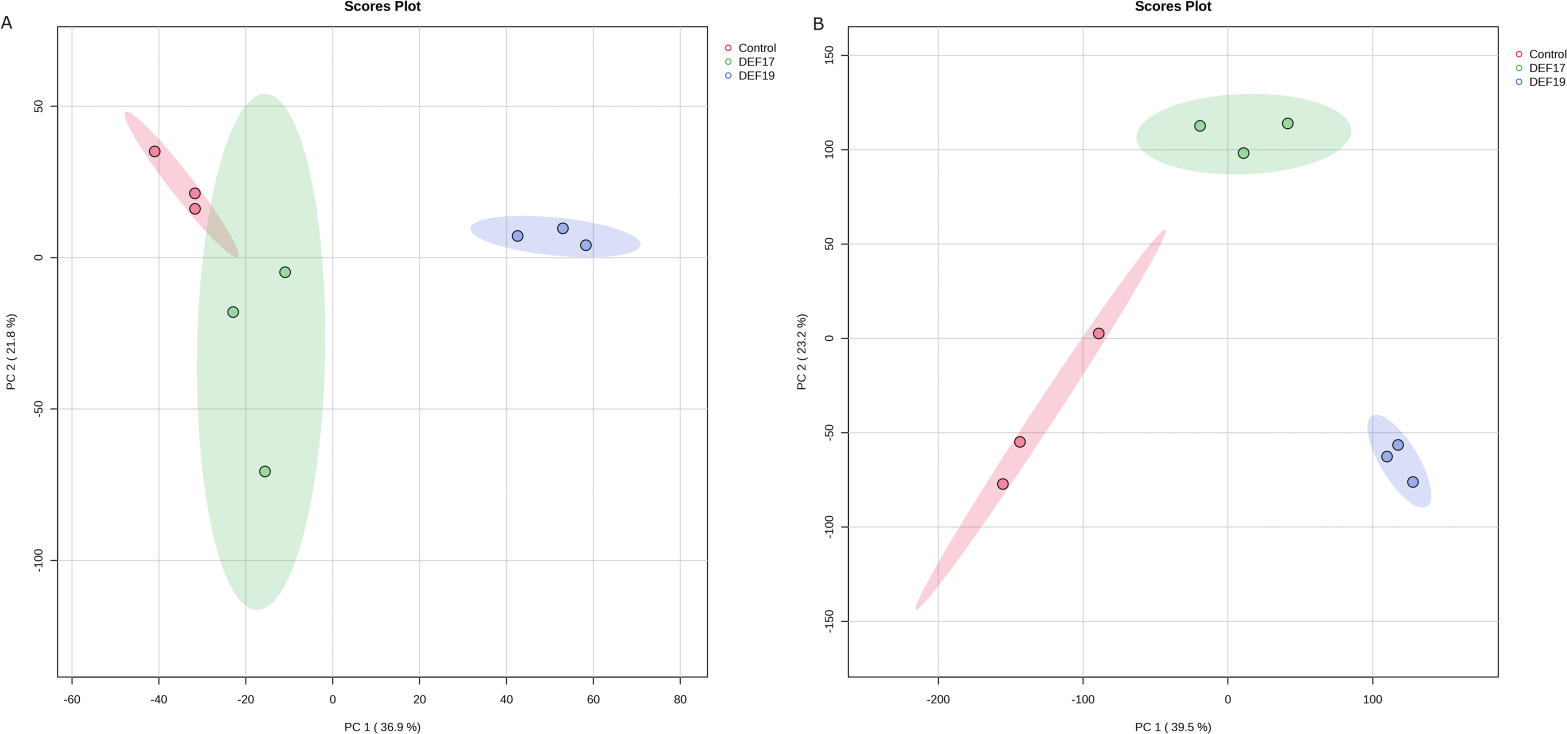
Principal components analysis (PCA) of root tissues **(A)** and root exudates **(B)**. In red: control group; In green: DEF17; In blue: DEF19.

Principal component analyses were conducted separately for root tissues and root exudates, reflecting their distinct metabolomic compartments. While DEF17-treated plants clustered closely with control plants at the root tissue level (**Figure 3a**), they formed a distinct cluster in root exudates (**Figure 3b**), indicating a preferential modulation of metabolite secretion rather than bulk root metabolism. This implies that DEF17 has a more limited but still unique effect on the roots and exudates, whilst DEF19 causes a more pronounced and widespread alteration of the studied metabolomes.

**Figure 4:**
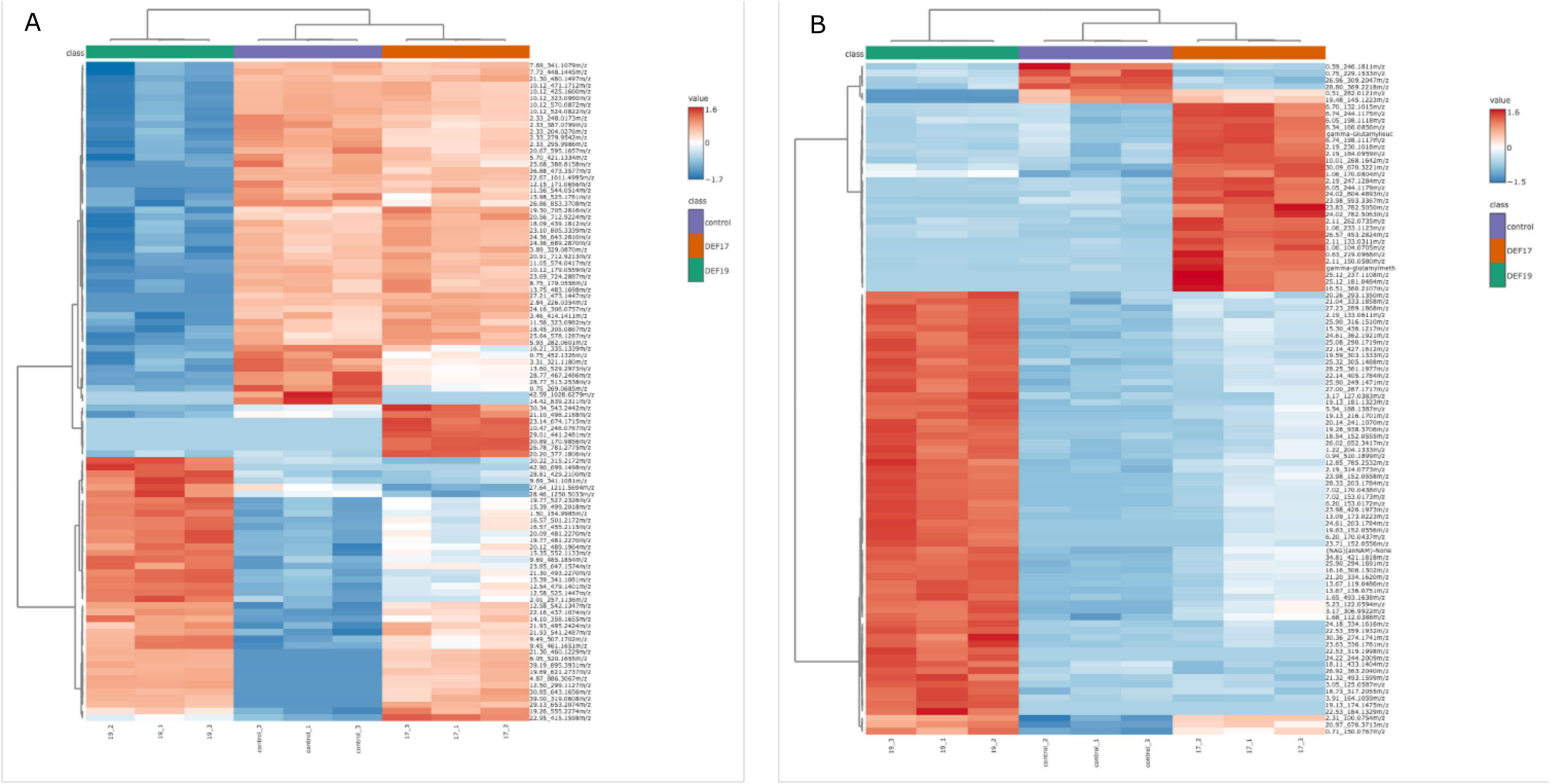
Heatmaps of the first 100 metabolites in root tissue **(A)** and in root exudates **(B)**. Green group: DEF19; Purple group: Control; Orange group:: DEF17.

The unique impact of inoculation with the different streptomycetes on the metabolomes is confirmed by hierarchical clustering. The samples from the inoculated seeds with either strain are separated from the controls, but the height of branches containing samples inoculated with DEF19 is higher than that of samples inoculated with DEF17 (**Figure 4a**). On the other hand, DEF17-treated plants exhibited a distinct and reproducible chemical fingerprint in root exudates, as revealed by the heatmap analysis (**Figure 4b**), characterized by treatment-specific patterns of relative metabolite abundance, suggesting a significant impact on secretion dynamics. As for root tissues, DEF19 induced the strongest response in tomato root exudates.

**Figure 5:**
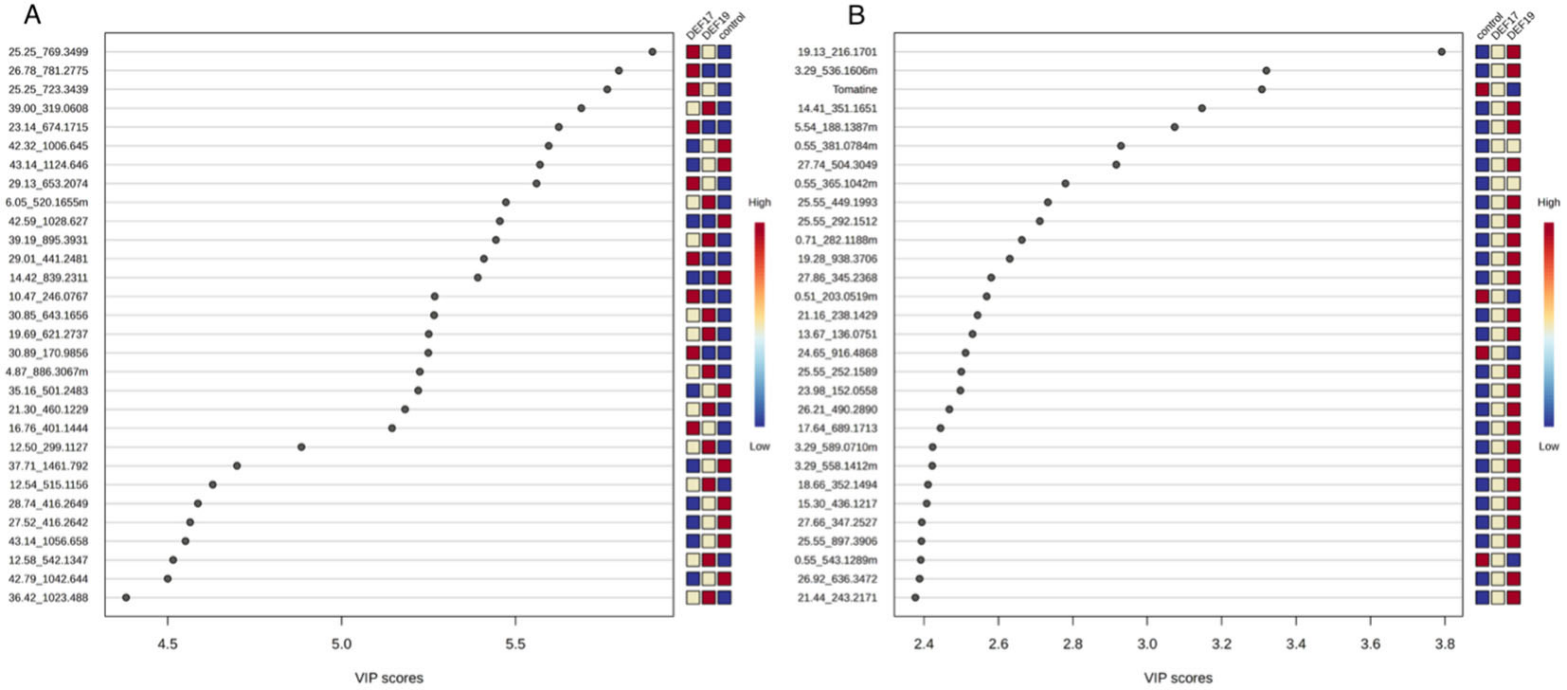
PLS-DA VIP scores for root tissues **(A)** and root exudates **(B)**.

In root tissues (**Figure 5a**), the top 30 discriminant features (VIP > 4.4) showed distinct accumulation patterns across treatments. In this analysis, most metabolites were more abundant in streptomycetes-treated samples than in control samples, but strain-specific effects are obvious, as seen already in **Figure 4**. The view in root exudates is different (**Figure 5b**); the majority of the high-VIP compounds are more abundant in exudates from DEF19-treated plants. These abundances are lowest in control-exudates, with DEF17-treated plants taking an intermediate abundance. Notably, tomatine showed higher abundance in control samples and was strongly reduced in DEF19. Since this decrease is much less pronounced in DEF17-exudates, these data show that there is a strain-specific alteration in the exudation of this defense-related metabolite.

Overall, these results are in line with those observed in the heatmap, indicating that *Streptomyces* spp. treatments, particularly DEF19, induced a different reprogramming in tomato root and root exudate metabolomes.

### 3.3 Strain-specific modulation and compartmentalization of tomato metabolites

Among the identified metabolites that change significantly depending on the treatment, a total of 22 metabolites were detected in both roots and exudates (**Supplementary Table S2**) showing different abundances depending on the sample type and treatment.

**Figure 6:**
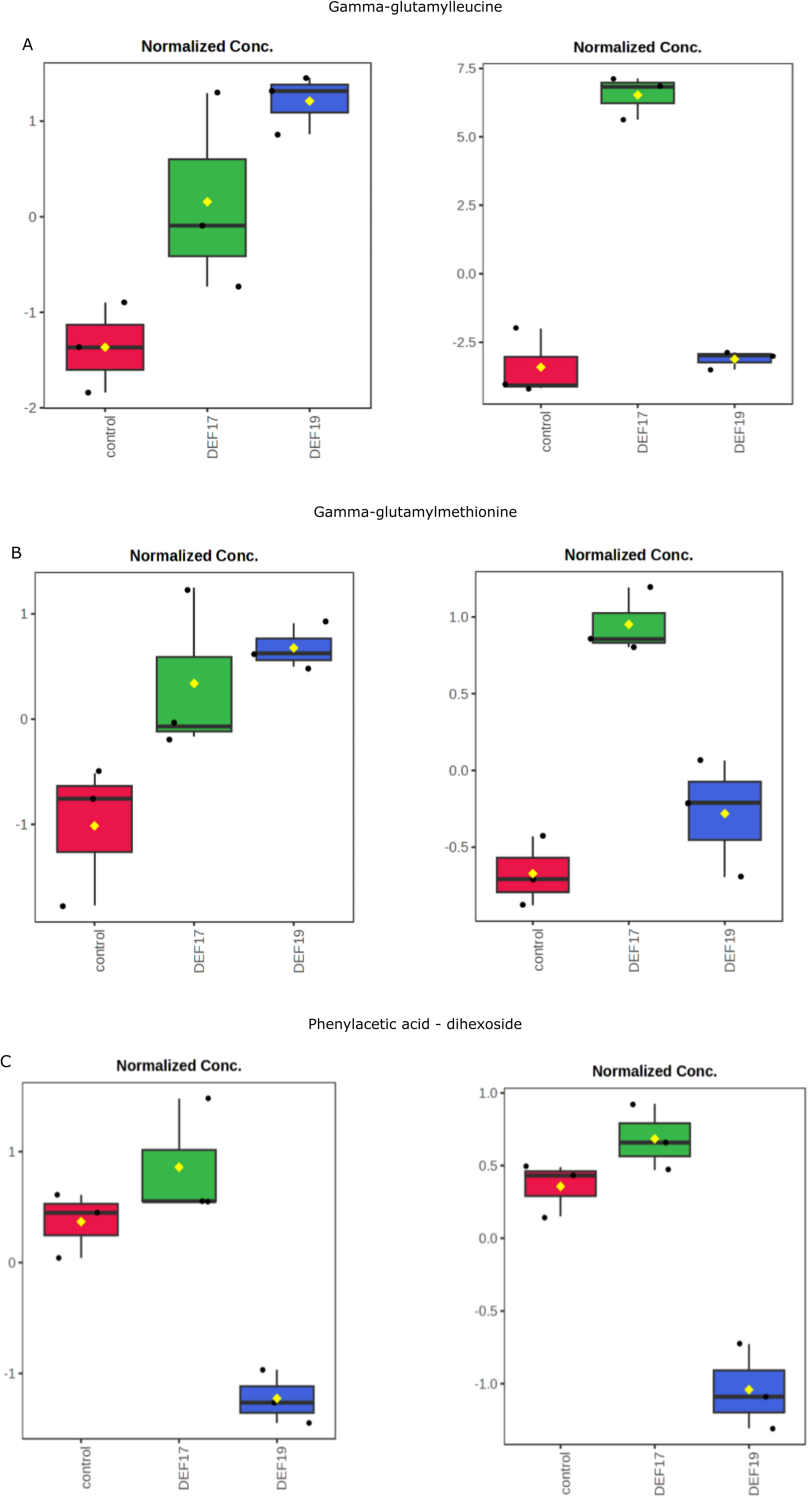
**(A)** Gamma-glutamyl leucine abundances in root tissues (left) and in root exudates (right); **(B)** Gamma-glutamyl methionine abundances in root tissues (left) and in root exudates (right); **(C)** PAA-dihexoside abundances in root tissues (left) and in root exudates (right) (Tukey’s HSD p > 0.05). Control plants (red), DEF17 (green) and DEF19 (blue) (Tukey’s HSD P< 0.05).

For instance, γ-glutamyl leucine (**Figure 6a**) exhibited clear compartment-specific patterns. In root tissues, DEF19-treated plants exhibited the highest concentrations, followed by DEF17-treated plants and the control. On the other hand, the highest concentrations were found in the root exudates of DEF17-treated plants. The abundance profile of γ-glutamyl methionine (**Figure 6b**) has the same patterns, being more abundant in roots of DEF19-treated plants and significantly increased in exudates of DEF17-treated plants compared to both DEF19 and control plants. Instead, phenylacetic acid dihexoside (PAA-dihex) (**Figure 6c**) showed similar patterns in both root tissues and root exudates with DEF17-treated plants showing significantly higher abundances in roots tissues and root exudates. Overall, these results show that the abundance of metabolites found in root tissues and root exudates is differently modulated by the two streptomycete strains in a compartment-specific manner.

**Figure 7:**
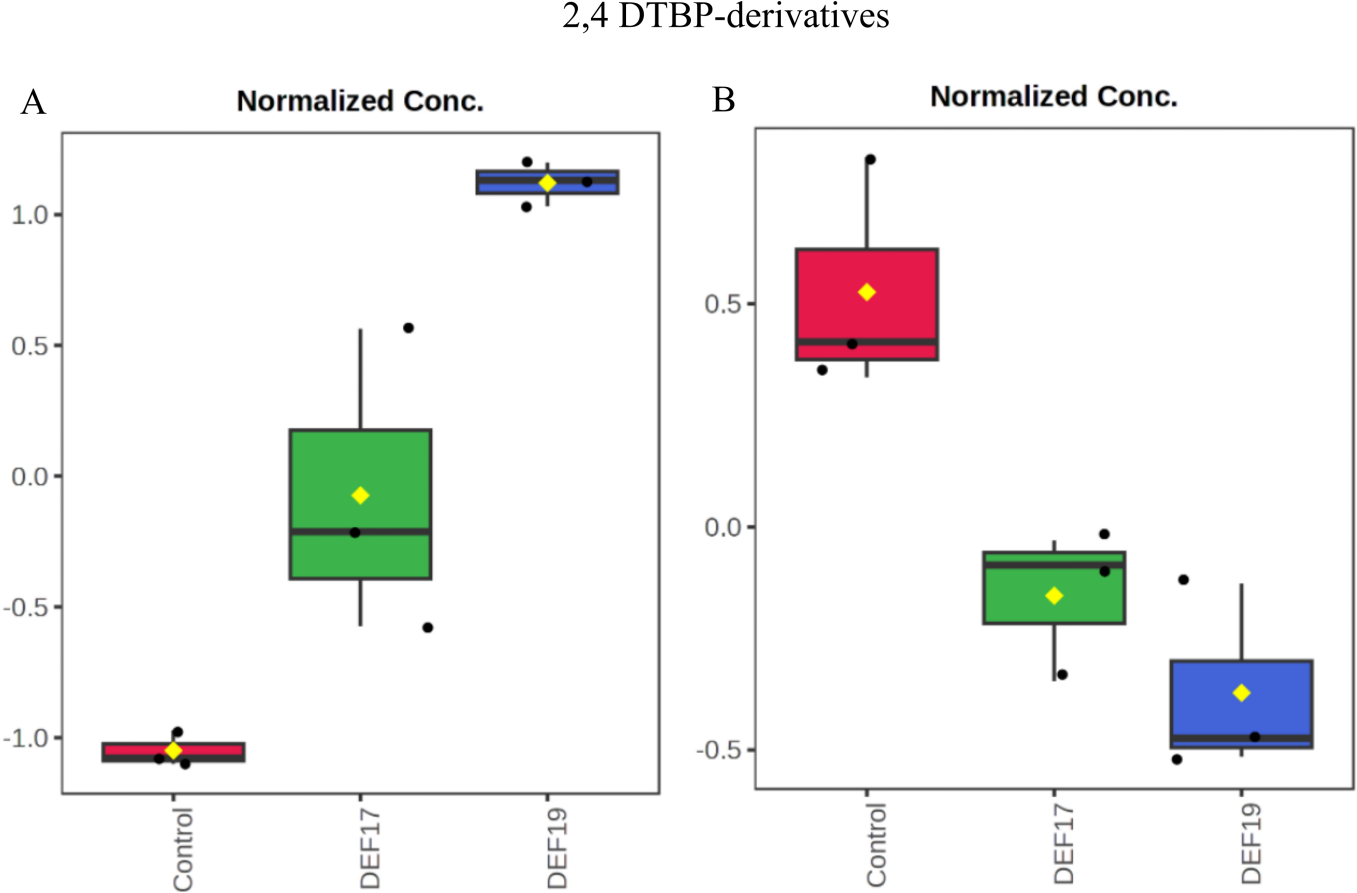
Abundances in root exudates of 2,4-DTBP Hex **(A)**; 2,4-DTBP HexHex **(B)** (Tukey’s HSD P< 0.05). Control plants (red), DEF17 (green) and DEF19 (blue).

In addition to these compartmentalization patterns, some finer modulation of the metabolome is observed. DEF19-treated plants showed a significantly higher signal for 2,4-di-tert-butylphenol hexoside (2,4-DTBP Hex), while the control group presented higher levels of a related derivative, 2,4-di-tert-butylphenol hex-hex (2,4-DTBP HexHex) (**Figures 7a, b**), with DEF17 showing in both cases at an intermediate abundance.

### 3.4 *In planta* pathogenesis assay

**Figure 8:**
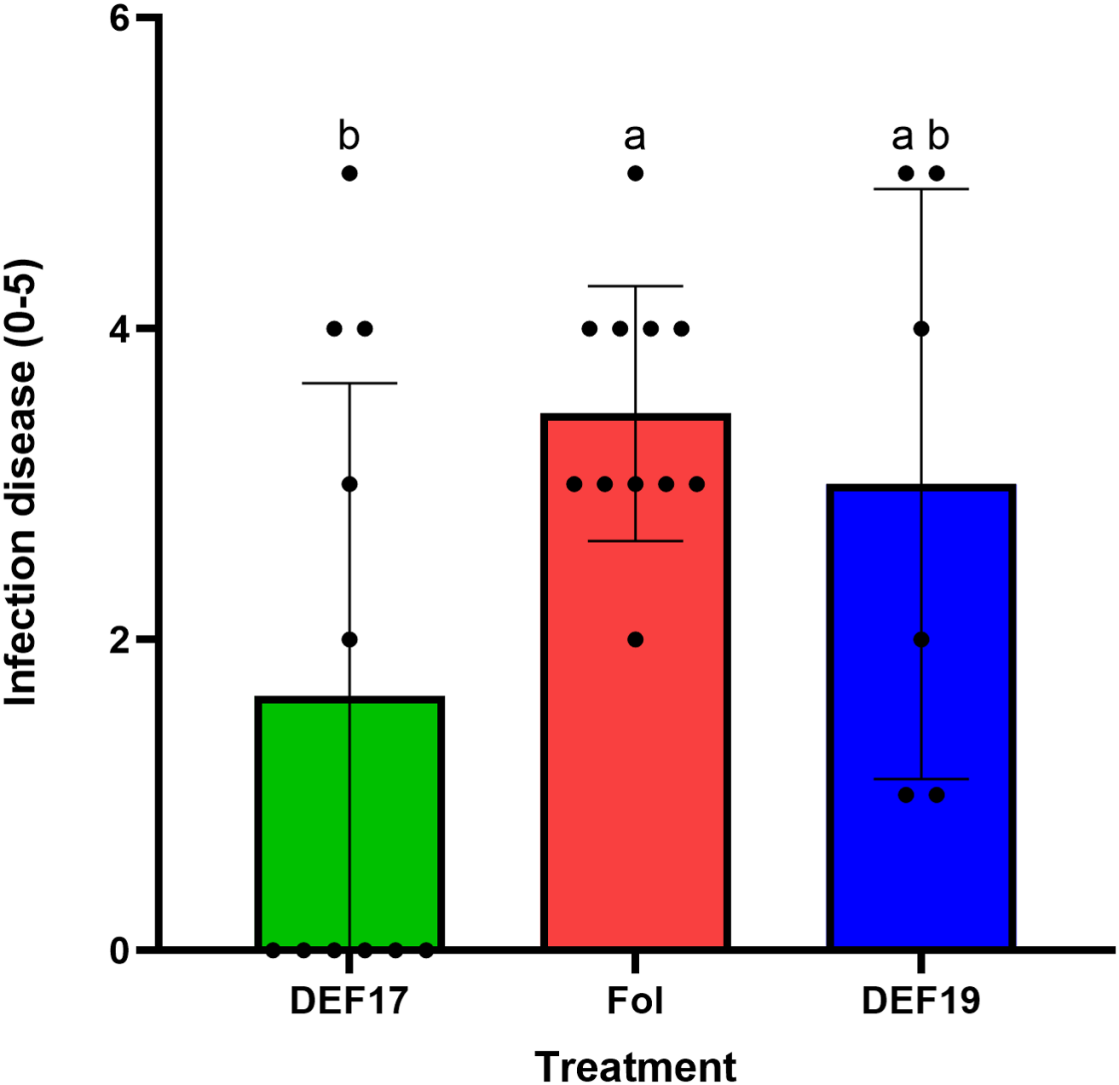
Disease severity of tomato plants (0–5 scale) following inoculation with Fol. Green) DEF17 seed treated plants inoculated with Fol, Red) Control plants inoculated with Fol, and Blue) DEF19 inoculated with Fol. Bars represent mean ± SD. Different letters indicate significant differences among treatments (Tukey’s HSD P< 0.05).

*In planta* assays revealed that DEF17 seed-treated plants showed significantly reduced disease severity compared to both control and DEF19 seed-treated plants, at 30 days post-inoculation (**Figure 8**; **Supplementary Figure S2**). On the other hand, control plants showed the higher severity scores, consistent with the known susceptibility of the ‘Moneymaker’ cultivar to Fol. In contrast, DEF19- seed-treated plants did not differ significantly from the infected control plants, indicating that this strain did not reduce significantly disease severity *in vivo*.

## 4 Discussion

This study demonstrates that distinct *Streptomyces* spp. strains can differentially modulate the tomato metabolome, both in root tissues and in secreted exudates. Metabolomic analyses revealed that DEF19 elicited more pronounced metabolome changes compared to DEF17, whose metabolome profile was more similar to that of untreated plants. Moreover, root vs root secretome-specific effects were also observed, highlighting the complexity of the plant–bacteria interactions and its effects on the host metabolism.

In plants, dipeptides can originate from different biosynthetic pathways and functions related to nitrogen storage and mobilization, antioxidants, signaling molecules, protein regulators, and modulators of plant–microbe interactions have been attributed (Agarwal et al., 2025; Nishioka et al., 2022; Solis-Ortiz et al., 2022). In this regard, dipeptides that contain γ-glutamyl are the only group of non-proteinogenic dipeptides in plants, and their biosynthesis is attributed and linked to GSH cycling and its fluctuations (Agarwal et al., 2025). Their biosynthesis depends on γ-glutamyl-Cys synthesis from L-Glu and L-Cys by γ-Glu–Cys synthetase (GCS), the rate-limiting enzyme in GSH pathway (Wang et al., 2017). Moreover, it has been observed that an increase in GSC led to an increase in γ-Glu–Cys conferring tolerance to abiotic stresses, such as heavy metals, like cadmium (He et al., 2015; Zhang et al., 2022a; Chhikara et al., 2024), drought and salt (Choe et al., 2013; Yang et al., 2024). The synthesis of other γ-glutamyl dipeptides depends on γ-glutamyl transpeptidase (GGT), in the GSH pathway, that transfers the γ-glutamyl residue from GSH to another proteinogenic or non-proteinogenic amino acid (Shaw et al., 2005). In this regard, Yu et al. (2024) noted that γ-Glu-Ala and GSH increased, leading to a reduction in γ-Glu-Cys after pumpkin seedlings were treated with tetracycline, suggesting that γ-Glu-Ala acts as antioxidant. Furthermore, the sulfur-containing dipeptide γ-Glu-Met acts as a ROS scavenger.

Furthermore, γ-glutamyl leucine was previously described by Westphal et al. (2019) as a stimulator of plant defense signaling in *Arabidopsis*, where it caused a weak induction of some MAMP-inducible genes and affected the refractory period to a second MAMP elicitation. However, γ-Glu-Leu was not considered a classical MAMP, since its activity was abolished by pH adjustment and its effects could be mimicked by extracellular acidification. In this regard, the pH from DEF17-treated exudates was lower compared to the other treatments. The higher abundance of γ-Glu-Leu in DEF17-treated plant exudates can thus be related to Fol phobotaxis. These results agree with Palmieri et al. (2020), who showed that rhizosphere acidification by *Rahnella aquatilis* through gluconic acid counteracts Fol-induced alkalinization, a known virulence mechanism. DEF17-treated plants showed significantly higher abundances of phenylacetic acid derivatives (PAA-dihexoside) in root exudates compared to DEF19-treated plants. Akram et al. (2016) observed that PAA secreted by *Bacillus fortis* acts as an inducer of systemic resistance (ISR) elicitor, up-regulating the biosynthesis of phenylpropanoid precursors and thus rerouting plant metabolism and inducing plant-defense mechanisms. On top of that, ISR activation by PAA led to suppression of *Fusarium* wilt in tomato plants, reducing disease index by up to 76% compared to infected control plants. However, the plant–bacteria interaction is a complex system in which defining the origin of the different metabolites, whether they are produced by the plant metabolism or by streptomycetes, is difficult to understand.

Metabolite profiling also revealed contrasting patterns for 2,4-di-tert-butylphenol (2,4-DTBP) derivatives. These compounds could be produced both by plants and streptomycetes with antimicrobial effects (Seenivasan et al., 2022; Kaari et al., 2023). However, in plants, 2,4-DTBP has also been shown to have potential auto-toxic effects as reported by Zhao et al. (2020). Furthermore, at high concentrations, 2,4-DTBP promotes the growth of fungal pathogens like *Fusarium* spp., thus reducing the populations of beneficial bacteria in the rhizosphere (Cui et al., 2022). In our experiments, DEF19-treated plants exhibited a marked increase in the monohexosylated form (2,4-DTBP hexoside), whereas control plants displayed higher levels of the dihexosylated derivative (2,4-DTBP hex-hex). The higher levels of the dihexosylated form in control plants likely reflect an effective detoxification strategy via full glycosylation, reducing the compound’s bioactivity (Zhang et al., 2022b). On the other hand, the monohexosylated form in DEF17-treated plants showed intermediate abundances between control and DEF19, suggesting a moderated detoxification response. In this regard, the prevalence of the monohexosyl derivative in DEF19 may reflect an incomplete detoxification, potentially altering the chemistry of the rhizosphere in ways that favor Fol chemotaxis. Given this, we hypothesize that the intermediate profile of DEF17-treated plants could maintain some deterrent activity while avoiding excessive autotoxicity, thus offering a balanced defense strategy.

Finally, this work emphasizes that different strains of *Streptomyces* sp. elicit unique metabolic responses in plants. When evaluating plant–microbe metabolic interactions, it is important to consider both spatial and functional complexity. This is demonstrated by the divergent metabolite profiles seen in DEF17 and DEF19 treatments, as well as the varying abundance of common molecules in various compartments. This redistribution is consistent with the concept that root exudation dynamics are highly plastic and species- or condition-specific, shaping rhizosphere chemistry and biological interactions (McLaughlin et al., 2023). Overall, these results are consistent with Mhlongo et al. (2020), who demonstrated that different rhizobacteria alter different classes of compounds in tomato roots and exudates, thus influencing rhizosphere composition and plant defense responses as demonstrated by *in planta* pathogenesis assay. Although DEF19 induced a broader metabolomic remodeling compared to DEF17, this did not translate into effective disease suppression, indicating that the extent of metabolome alteration is not directly proportional to Fol biocontrol. In contrast, the more targeted metabolic modulation induced by DEF17 was associated with a stronger reduction in Fol pathogenicity.

To conclude, the application of streptomycetes as seed treatment, differently from Akram et al. (2015), who drenched tomato plants with the applications of *B. fortis* cell free culture filtrate (CFCF) containing bacterial secondary metabolites, inherently leads to difficulties in defining the origin of the identified metabolites. Factors such as bacterial mimicry during plant-microbe interactions (Stringlis et al., 2018) but also metabolic activities of the microbial component (modification, degradation) on exudated metabolites may directly influence the metabolic profile. However, our results indicate that DEF17 seed treatment can be exploited as a promising biocontrol agent in tomato against Fol despite the absence of direct *in vitro* antifungal activity. The observed reduction in disease severity is consistent with an exudate-mediated and plant-dependent mode of action, underscoring the importance of evaluating biocontrol candidates *in planta* and considering indirect mechanisms based on host metabolic modulation rather than relying solely on antagonistic assays. Future studies will be required to further elucidate the molecular and physiological mechanisms underlying DEF17-mediated disease suppression, including the origin, stability, and bioactivity of key metabolites identified in root exudates. Approaches such as stable isotope labelling, as demonstrated by Pang et al. (2018), could enable discrimination between plant- and microbe-derived compounds. In addition, targeted extraction and testing of selected metabolites, together with validation under greenhouse and field conditions, will be essential to assess the translational potential of DEF17 as a sustainable biocontrol agent against *Fusarium* wilt.

## Data Availability

The original contributions presented in the study are included in the article/**Supplementary Material**. Further inquiries can be directed to the corresponding author.

## References

[B1] Agarwal P. Fischer H. D. Camalle M. D. Skirycz A. (2025). Not to be overlooked: dipeptides and their role in plant stress resilience. J. Exp. Bot. 76, 5738–5747. doi: 10.1093/jxb/eraf311, PMID: 40628532 PMC12516527

[B2] Akram M. Asghar H. Zahir Z. Ahmad H. Hussain M. B. (2015). Isolation and screening of beneficial bacteria to ameliorate drought stress in wheat. Soil & Environment 76, 5738–5747.

[B3] Akram W. Anjum T. Ali B. (2016). Phenylacetic Acid Is ISR Determinant Produced by *Bacillus fortis* IAGS162, Which Involves Extensive Re-modulation in Metabolomics of Tomato to Protect against Fusarium Wilt. Front. Plant Sci. 7. doi: 10.3389/fpls.2016.00498, PMID: 27148321 PMC4835451

[B4] Barka E. A. Vatsa P. Sanchez L. Gaveau-Vaillant N. Jacquard C. Meier-Kolthoff J. P. . (2016). Taxonomy, physiology, and natural products of actinobacteria. Microbiol. Mol. Biol. Rev. 80, 1–43. doi: 10.1128/MMBR.00019-15, PMID: 26609051 PMC4711186

[B5] Canarini A. Kaiser C. Merchant A. Richter A. Wanek W. (2019). Root exudation of primary metabolites: mechanisms and their roles in plant responses to environmental stimuli. Front. Plant Sci. 10. doi: 10.3389/fpls.2019.00157, PMID: 30881364 PMC6407669

[B6] Chhikara S. Singh Y. Long S. Minocha R. Musante C. White J. C. . (2024). Overexpression of bacterial γ-glutamyl cysteine synthetase increases toxic metal(loid)s tolerance and accumulation in *Crambe abyssinica*. Plant Cell Rep. 43, 270. doi: 10.1007/s00299-024-03351-3, PMID: 39443376 PMC11660394

[B7] Choe Y.-H. Kim Y.-S. Kim I.-S. Bae M.-J. Lee E.-J. Kim Y.-H. . (2013). Homologous expression of γ-glutamylcysteine synthetase increases grain yield and tolerance of transgenic rice plants to environmental stresses. J. Plant Physiol. 170, 610–618. doi: 10.1016/j.jplph.2012.12.002, PMID: 23294545

[B8] Colombo E. M. Kunova A. Pizzatti C. Saracchi M. Cortesi P. Pasquali M. (2019). Selection of an Endophytic *Streptomyces* sp. Strain DEF09 From Wheat Roots as a Biocontrol Agent Against *Fusarium graminearum*. Front. Microbiol. 10. doi: 10.3389/fmicb.2019.02356, PMID: 31681219 PMC6798073

[B9] Corkley I. Fraaije B. Hawkins N. (2022). Fungicide resistance management: Maximizing the effective life of plant protection products. Plant Pathol. 71, 150–169. doi: 10.1111/ppa.13467, PMID: 41744481

[B10] Cui J. Zhang E. Zhang X. Wang Q. Liu Q. (2022). Effects of 2,4-di-tert-butylphenol at different concentrations on soil functionality and microbial community structure in the Lanzhou lily rhizosphere. Appl. Soil Ecol. 172, 104367. doi: 10.1016/j.apsoil.2021.104367, PMID: 41760527

[B11] Dean R. Van Kan J. A. L. Pretorius Z. A. Hammond-Kosack K. E. Di Pietro A. Spanu P. D. . (2012). The Top 10 fungal pathogens in molecular plant pathology. Mol. Plant Pathol. 13, 414–430. doi: 10.1111/j.1364-3703.2011.00783.x, PMID: 22471698 PMC6638784

[B12] Halime S. Renaut J. Zimmer S. Heidt H. Jacquard C. Sergeant K. (2025). Comparative metabolomic profiling of *Lupinus albus* and *L. angustifolius* harvest residues: exploring chemical diversity and valorization potential. Front. Plant Sci. 16. doi: 10.3389/fpls.2025.1617634, PMID: 40692666 PMC12277371

[B13] He J. Li H. Ma C. Zhang Y. Polle A. Rennenberg H. . (2015). Overexpression of bacterial γ-glutamyl cysteine synthetase mediates changes in cadmium influx, allocation and detoxification in poplar. New Phytol. 205, 240–254. doi: 10.1111/nph.13013, PMID: 25229726

[B14] Kaari M. Joseph J. Manikkam R. Kalyanasundaram R. Sivaraj A. Anbalmani S. . (2023). A Novel Finding: 2,4-Di-tert-butylphenol from *Streptomyces bacillaris* ANS2 Effective Against *Mycobacterium tuberculosis* and Cancer Cell Lines. Appl. Biochem. Biotechnol. 195, 6572–6585. doi: 10.1007/s12010-023-04403-2, PMID: 36881320

[B15] Korenblum E. Dong Y. Szymanski J. Panda S. Jozwiak A. Massalha H. . (2020). Rhizosphere microbiome mediates systemic root metabolite exudation by root-to-root signaling. Proc. Natl. Acad. Sci. U.S.A. 117, 3874–3883. doi: 10.1073/pnas.1912130117, PMID: 32015118 PMC7035606

[B16] Kunova A. Bonaldi M. Saracchi M. Pizzatti C. Chen X. Cortesi P. (2016). Selection of Streptomyces against soil borne fungal pathogens by a standardized dual culture assay and evaluation of their effects on seed germination and plant growth. BMC Microbiol. 16, 272. doi: 10.1186/s12866-016-0886-1, PMID: 27829359 PMC5103511

[B17] Liu K. Newman M. McInroy J. A. Hu C.-H. Kloepper J. W. (2017). Selection and assessment of plant growth-promoting rhizobacteria for biological control of multiple plant diseases. Phytopathology® 107, 928–936. doi: 10.1094/PHYTO-02-17-0051-R, PMID: 28440700

[B18] Lombardi N. Vitale S. Turrà D. Reverberi M. Fanelli C. Vinale F. . (2018). Root exudates of stressed plants stimulate and attract *Trichoderma* soil fungi. MPMI 31, 982–994. doi: 10.1094/MPMI-12-17-0310-R, PMID: 29547355

[B19] Mahmud A. A. Upadhyay S. K. Srivastava A. K. Bhojiya A. A. (2021). Biofertilizers: A Nexus between soil fertility and crop productivity under abiotic stress. Curr. Res. Environ. Sustainability 3, 100063. doi: 10.1016/j.crsust.2021.100063, PMID: 41760527

[B20] Marlatt M. L. (1996). Two genetically distinct populations of *Fusarium oxysporum* f. sp. *lycopersici* race 3 in the United States. Plant Dis. 80, 1336. doi: 10.1094/PD-80-1336, PMID: 40211709

[B21] Mattei V. Motta A. Saracchi M. Kunova A. Cortesi P. Pizzatti C. . (2022). Wheat Seed Coating with *Streptomyces* sp. strain DEF39 spores protects against *Fusarium* Head Blight. Microorganisms 10, 1536. doi: 10.3390/microorganisms10081536, PMID: 36013954 PMC9415289

[B22] McLaughlin S. Zhalnina K. Kosina S. Northen T. R. Sasse J. (2023). The core metabolome and root exudation dynamics of three phylogenetically distinct plant species. Nat Commun 14, 1649. doi: 10.1038/s41467-023-37164-x, PMID: 36964135 PMC10039077

[B23] Mhlongo M. I. Piater L. A. Steenkamp P. A. Labuschagne N. Dubery I. A. (2020). Metabolic profiling of PGPR-treated tomato plants reveal priming-related adaptations of secondary metabolites and aromatic amino acids. Metabolites 10, 210. doi: 10.3390/metabo10050210, PMID: 32443694 PMC7281251

[B24] Michielse C. B. Rep M. (2009). Pathogen profile update: *Fusarium oxysporum*. Mol. Plant Pathol. 10, 311–324. doi: 10.1111/j.1364-3703.2009.00538.x, PMID: 19400835 PMC6640313

[B25] Nishioka T. Suga H. Shimizu M. (2022). The stimulation of indigenous bacterial antagonists by γ-glutamyl-S-Allyl-l-cysteine increases soil suppressiveness to *Fusarium* wilt. Appl. Environ. Microbiol. 88, e01554–e01522. doi: 10.1128/aem.01554-22, PMID: 36445356 PMC9765066

[B26] Palmieri D. Vitale S. Lima G. Di Pietro A. Turrà D. (2020). A bacterial endophyte exploits chemotropism of a fungal pathogen for plant colonization. Nat. Commun. 11, 5264. doi: 10.1038/s41467-020-18994-5, PMID: 33067433 PMC7567819

[B27] Pang Q. Zhang T. Wang Y. Kong W. Guan Q. Yan X. . (2018). Metabolomics of early-stage plant cell–microbe interaction using stable isotope labeling. Front. Plant Sci. 9. doi: 10.3389/fpls.2018.00760, PMID: 29922325 PMC5996122

[B28] Panth M. Hassler S. C. Baysal-Gurel F. (2020). Methods for management of soilborne diseases in crop production. Agriculture 10, 16. doi: 10.3390/agriculture10010016, PMID: 41725453

[B29] Seenivasan A. Manikkam R. Kaari M. Sahu A. K. Said M. Dastager S. G. (2022). 2,4-Di-tert-butylphenol (2,4-DTBP) purified from *Streptomyces* sp. KCA1 from *Phyllanthus niruri*: Isolation, characterization, antibacterial and anticancer properties. J. King Saud Univ. - Sci. 34, 102088. doi: 10.1016/j.jksus.2022.102088, PMID: 41760527

[B30] Shaw M. L. Pither-Joyce M. D. McCallum J. A. (2005). Purification and cloning of a γ-glutamyl transpeptidase from onion (*Allium cepa*). Phytochemistry 66, 515–522. doi: 10.1016/j.phytochem.2005.01.017, PMID: 15721943

[B31] Solis-Ortiz C. S. Gonzalez-Bernal J. Kido-Díaz H. A. Peña-Uribe C. A. López-Bucio J. S. López-Bucio J. . (2022). Bacterial cyclodipeptides elicit *Arabidopsis thaliana* immune responses reducing the pathogenic effects of *Pseudomonas aeruginosa* PAO1 strains on plant development. J. Plant Physiol. 275, 153738. doi: 10.1016/j.jplph.2022.153738, PMID: 35690030

[B32] Stringlis I. A. Zhang H. Pieterse C. M. J. Bolton M. D. de Jonge R. (2018). Microbial small molecules – weapons of plant subversion. Natural Product Rep. 35, 410–433. doi: 10.1039/C7NP00062F, PMID: 29756135

[B33] Turrà D. El Ghalid M. Rossi F. Di Pietro A. (2015). Fungal pathogen uses sex pheromone receptor for chemotropic sensing of host plant signals. Nature 527, 521–524. doi: 10.1038/nature15516, PMID: 26503056

[B34] Upadhyay S. K. Srivastava A. K. Rajput V. D. Chauhan P. K. Bhojiya A. A. Jain D. . (2022). Root exudates: mechanistic insight of plant growth promoting rhizobacteria for sustainable crop production. Front. Microbiol. 13. doi: 10.3389/fmicb.2022.916488, PMID: 35910633 PMC9329127

[B35] Viaene T. Langendries S. Beirinckx S. Maes M. Goormachtig S. (2016). *Streptomyces* as a plant’s best friend? FEMS Microbiol. Ecol. 92, fiw119. doi: 10.1093/femsec/fiw119, PMID: 27279415

[B36] Vives-Peris V. de Ollas C. Gómez-Cadenas A. Pérez-Clemente R. M. (2020). Root exudates: from plant to rhizosphere and beyond. Plant Cell Rep. 39, 3–17. doi: 10.1007/s00299-019-02447-5, PMID: 31346716

[B37] Vurukonda S. S. K. P. Giovanardi D. Stefani E. (2018). Plant growth promoting and biocontrol activity of *Streptomyces* spp. as endophytes. Int. J. Mol. Sci. 19, 952. doi: 10.3390/ijms19040952, PMID: 29565834 PMC5979581

[B38] Wang S. Cao Z. Chen Y. Wu R. (2017). Enhancement of Cadmium Tolerance in Transgenic Tobacco Plants by Transferring a ?-glutamylcysteine Synthetase Gene. Journal of Agronomy and Agricultural Aspects 2. Available at: https://www.gavinpublishers.com/article/view/enhancement-of-cadmium-tolerance-in-transgenic-tobacco-plants-by-transferring-a-y-glutamylcysteine-synthetase-gene (Accessed September 4, 2025).

[B39] Wankhade A. Wilkinson E. Britt D. W. Kaundal A. (2025). A review of plant–microbe interactions in the rhizosphere and the role of root exudates in microbiome engineering. Appl. Sci. 15, 7127. doi: 10.3390/app15137127, PMID: 41725453

[B40] Westphal L. Strehmel N. Eschen-Lippold L. Bauer N. Westermann B. Rosahl S. . (2019). pH effects on plant calcium fluxes: lessons from acidification-mediated calcium elevation induced by the γ-glutamyl-leucine dipeptide identified from *Phytophthora infestans*. Sci. Rep. 9, 4733. doi: 10.1038/s41598-019-41276-0, PMID: 30894659 PMC6426842

[B41] Yang Z. Wang Y. Cheng Q. Zou X. Yang Y. Li P. . (2024). Overexpression of sweet potato glutamylcysteine synthetase (IbGCS) in *Arabidopsis* confers tolerance to drought and salt stresses. J. Plant Res. 137, 669–683. doi: 10.1007/s10265-024-01548-x, PMID: 38758249

[B42] Yu J. Han T. Hou Y. Zhao J. Zhang H. Wang X. . (2024). Integrated transcriptomic, proteomic and metabolomic analysis provides new insights into tetracycline stress tolerance in pumpkin. Environ. pollut. 340, 122777. doi: 10.1016/j.envpol.2023.122777, PMID: 37863256

[B43] Zhang W. Wang S. Yang J. Kang C. Huang L. Guo L. (2022a). Glycosylation of plant secondary metabolites: Regulating from chaos to harmony. Environ. Exp. Bot. 194, 104703. doi: 10.1016/j.envexpbot.2021.104703, PMID: 41760527

[B44] Zhang X. Zhang L. Chen L. Lu Y. An Y. (2022b). Ectopic expression γ-glutamyl cysteine synthetase of *Vicia sativa* increased cadmium tolerance in *Arabidopsis*. Gene 823, 146358. doi: 10.1016/j.gene.2022.146358, PMID: 35202731

[B45] Zhao F. Wang P. Lucardi R. D. Su Z. Li S. (2020). Natural sources and bioactivities of 2,4-Di-Tert-Butylphenol and its analogs. Toxins (Basel) 12, 35. doi: 10.3390/toxins12010035, PMID: 31935944 PMC7020479

[B46] Zhao X. Hou D. Xu J. Wang K. Hu Z. (2022). Antagonistic activity of fungal strains against *Fusarium* crown rot. Plants 11, 255. doi: 10.3390/plants11030255, PMID: 35161236 PMC8838148

