# Correction: Differential modulation of tomato root exudates by *Streptomyces* strains underlies contrasting control of *Fusarium oxysporum* f. sp. *lycopersici*

**DOI:** 10.3389/fpls.2026.1829345

**Published:** 2026-03-19

**Authors:** Frontiers Production Office

**Affiliations:** Frontiers Media SA, Lausanne, Switzerland

**Keywords:** bacteria plant interactions, biological control, root exudate, chemotropism, *Streptomyces* - tomato - fungi, untarget metabolomics, LC-MS/MS

The **Results** section of the Abstract was replaced with a section of unrelated text. The original paragraph stated “This study indicates that herbal formulas that could regulate the composition and proportion of gut microbiota have a positive effect in three stages (perioperative, postoperative, and advanced) of GC and CRC. They could promote the recovery of postoperative gastrointestinal function, increase tumor response, improve performance status, and reduce the incidence of adverse events. Herbal formulas exerted anti-cancer efficacy through multiple mechanisms and pathways; among them, the regulation of gut microbiota has not been paid enough attention. To further support the conclusion and better understand the role of gut microbiota in the treatment of GCand CRC, more rigorously designed, large-scale, and multicenter RCTs that focus on herbal formulas and gut microbiota are needed in the future.” The correct paragraph states “In dual culture assays, DEF19 inhibited Fol growth, whereas DEF17 did not. On the other hand, exudates derived from DEF17-seed treated plant showed to be more acidic, thus inducing Fol phobotaxis compared to both DEF19 and control plants. Also, DEF17-seed treated plants significantly reduced disease severity in planta. Metabolomics showed strain- and compartment- specific remodeling with DEF19 affecting it mostly. On the other hand, DEF17 showed a distinct exudate fingerprint enriched in g-glutamyl dipeptides and phenylacetic-acid conjugates, with an interaction-induced differential glycosylation of 2,4-di-tert-butylphenol”.

The original version of this article has been updated.

