# Supplementary material for: Differential modulation of tomato root exudates by Streptomyces strains underlies contrasting control of Fusarium oxysporum f. sp. lycopersici
Source: Front Plant Sci. 2026 Mar 4;17:1759226. doi: 10.3389/fpls.2026.1759226 (PMC12995628; doi:10.3389/fpls.2026.1759226)
Supplement: Supplementary file 1 [file DataSheet1.pdf]

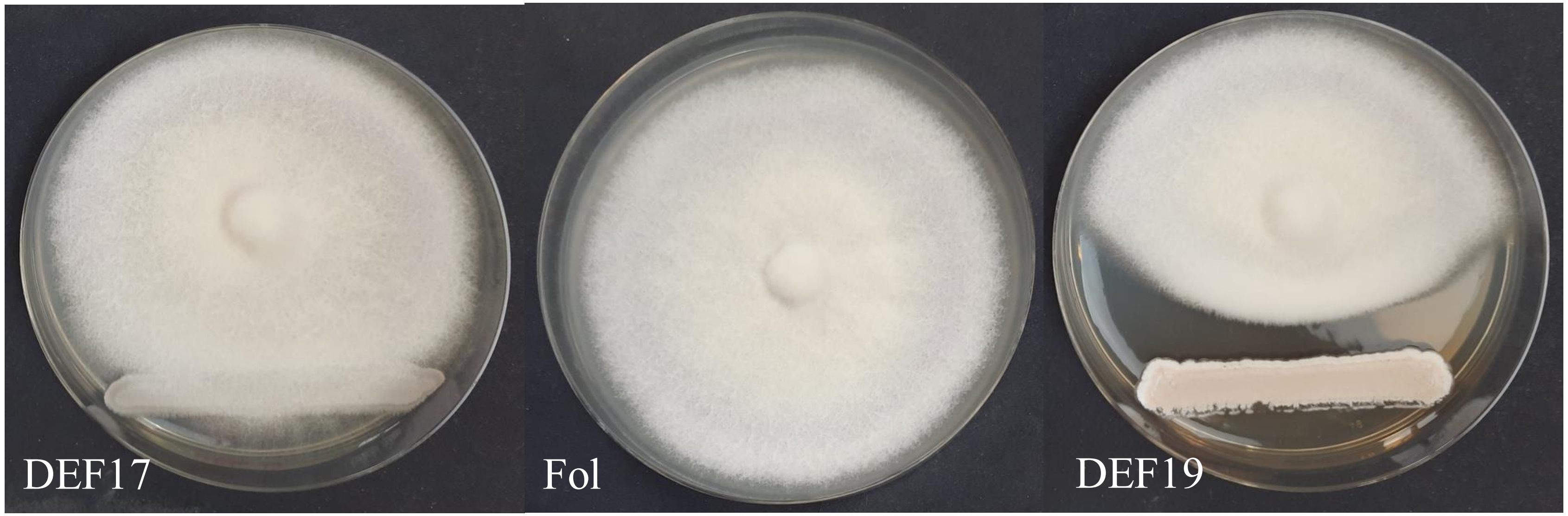

Fig S1: *In vitro* dual culture assay. A) Fol inhibition against DEF17; B) Control plate containing only Fol; Fol inhibition against DEF19

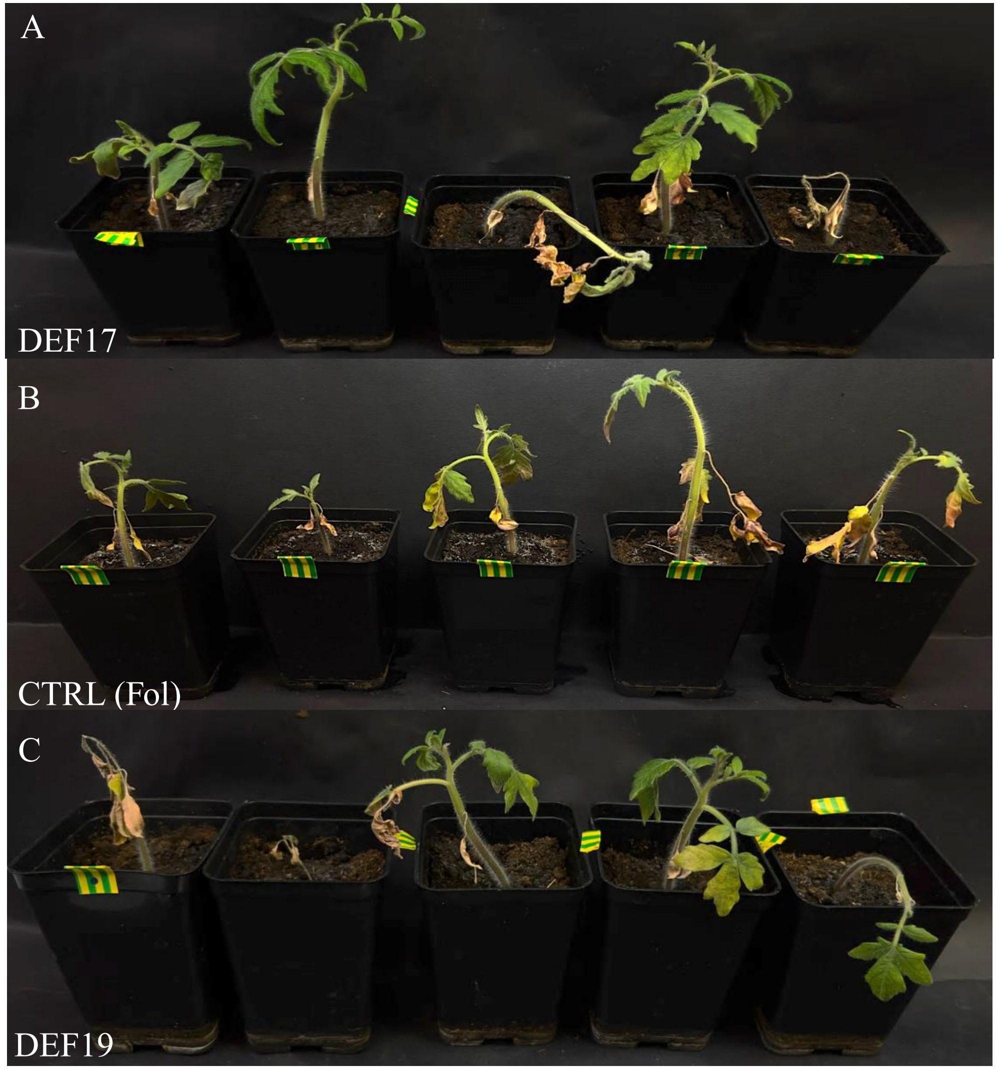

Fig S2: In plant pathogenesis assay. A) DEF17 seed treated plant + Fol; B) Control plants + Fol; C) DEF19 seed treated plants + Fol

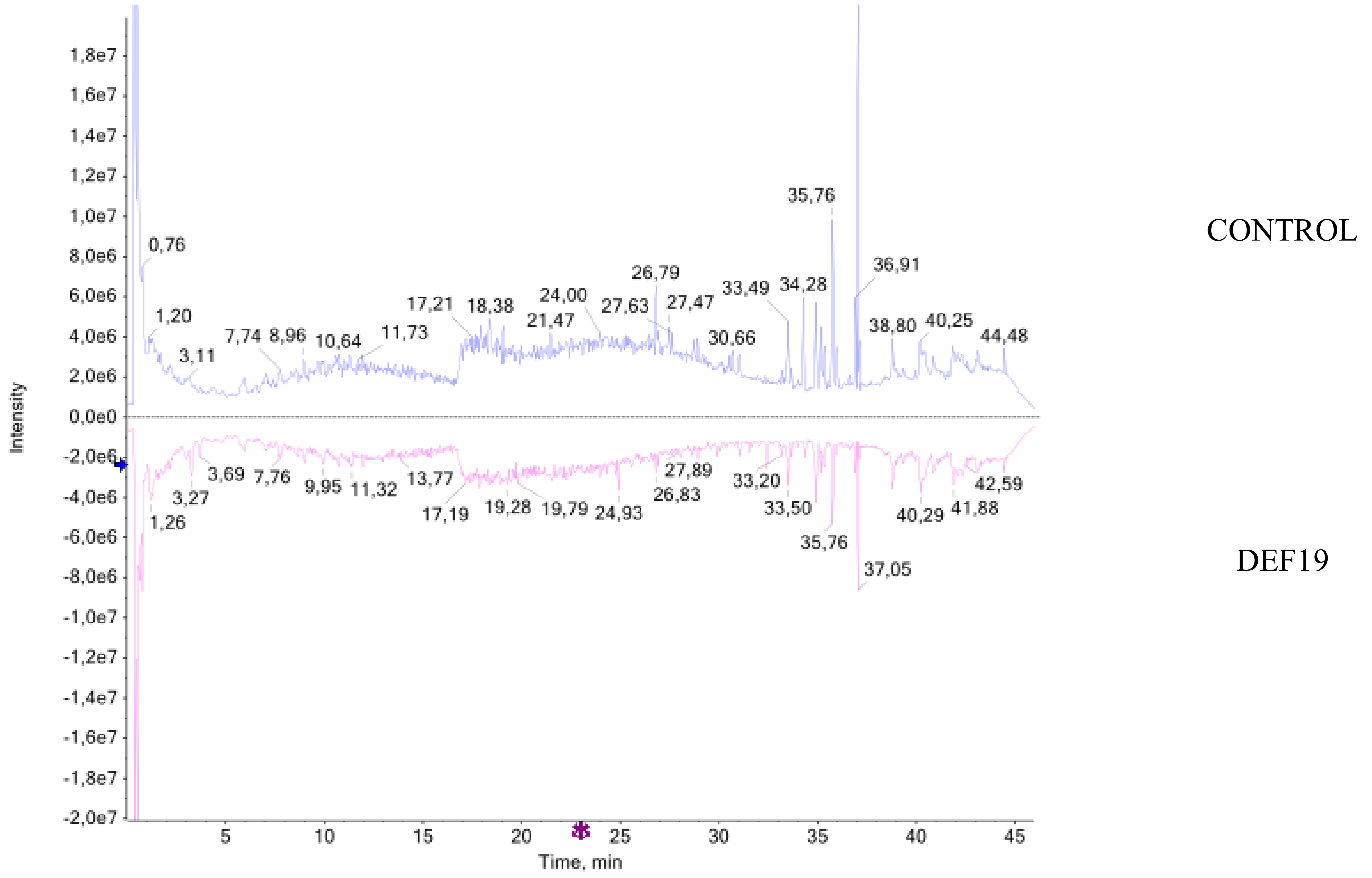

Fig S3: A) Exudates chromatogram overlay up) control; down) DEF19

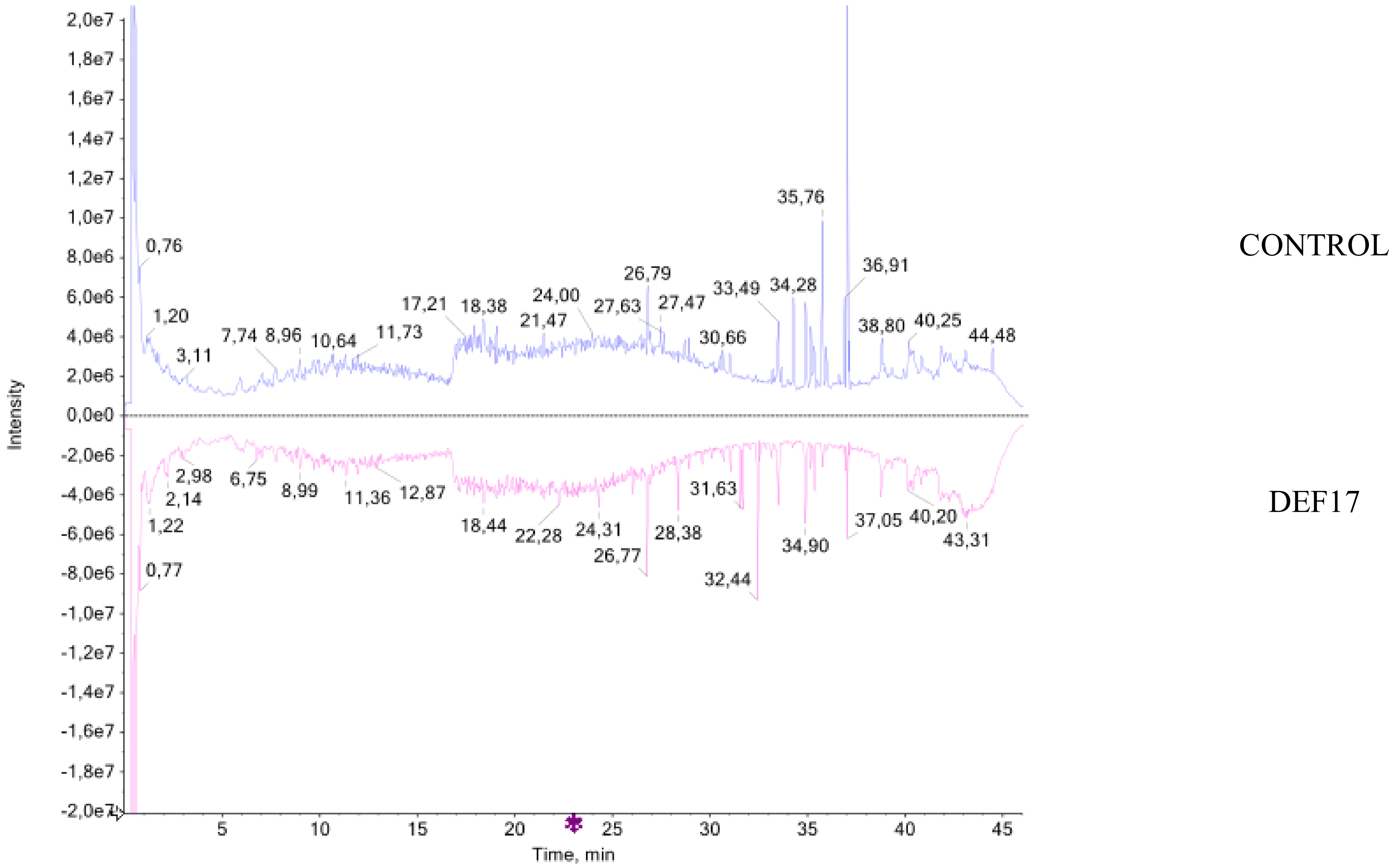

Fig S3: B) Exudates chromatogram overlay up) control; down) DEF17

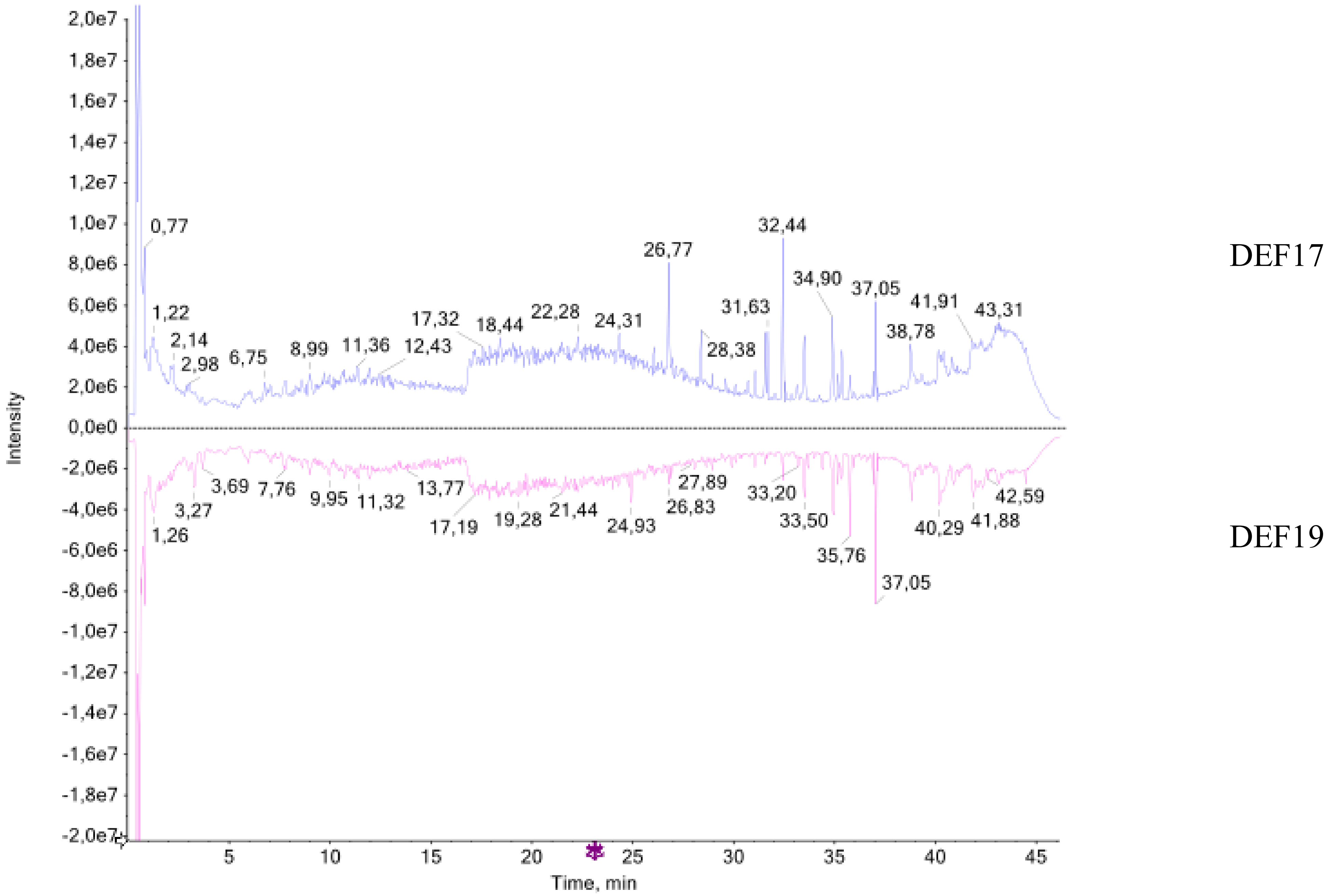

Fig S3: C) Exudates chromatogram overlay up) DEF17; down) DEF19
